# Oncogenic driver genes and tumor microenvironment determine the type of liver cancer

**DOI:** 10.1038/s41419-020-2509-x

**Published:** 2020-05-04

**Authors:** Gang Wang, Qian Wang, Ning Liang, Hongyuan Xue, Tao Yang, Xuguang Chen, Zhaoyan Qiu, Chao Zeng, Tao Sun, Weitang Yuan, Chaoxu Liu, Zhangqian Chen, Xianli He

**Affiliations:** 1 Department of General Surgery, The 74th Group Army Hospital, Guangzhou, 510220 China; 2 Department of General Surgery, Tangdu Hospital, Air Force Military Medical University, Xi’an, 710032 Shaanxi China; 3 Department of Anorectal Surgery, First Affiliated Hospital, Zhengzhou University, Zhengzhou, 450052 China; 4 Department of General Surgery, The 75th Group Army Hospital, Dali, 671000 China; 5 Department of General Surgery, Huashan North Hospital, Fudan University, Shanghai, 201907 China; 6 Department of Pain Treatment, Tangdu Hospital, Air Force Military Medical University, Xi’an, 710032 Shanxi China; 7 Department of Dermatology, Dermatology Hospital of Southern Medical University, Guangzhou, 510091 China; 8 Department of General Surgery, Chinese PLA General Hospital, Beijing, China; 9 Department of Cardiology, The 74th Group Army Hospital, Guangzhou, 510318 China; 10 Departmentof Neurosurgery, First Affiliated Hospital, Zhengzhou University, Zheng zhou, 450052 China; 11 Department of Anorectal Surgery, The First Affiliated Hospital of Zhejiang University, Hangzhou, 310003 China; 12 Department of Infectious Diseases, Xijing Hospital, Air Force Military Medical University, Xi’an, 710032 Shaanxi China; 13 State key Laboratory of Cancer Biology, National Clinical Research Center for Digestive Diseases and Xijing Hospital of Digestive Diseases, Air Force Military Medical University, Xi’an, 710032 Shaanxi China

**Keywords:** Liver cancer, Liver cancer

## Abstract

Primary liver cancer (PLC) may be mainly classified as the following four types: hepatocellular carcinoma (HCC), intrahepatic cholangiocarcinoma (ICC), hepatoblastoma (HB), and combined hepatocellular carcinoma and intrahepatic cholangiocarcinoma (cHCC-ICC). The majority of PLC develops in the background of tumor microenvironment, such as inflammatory microenvironments caused by viral hepatitis, alcoholic or nonalcoholic steatohepatitis, carbon tetrachloride (CCl_4_), 3,5-diethoxycarbonyl-1,4-dihydrocollidine (DDC), and necroptosis-associated hepatic cytokine microenvironment caused by necroptosis of hepatocytes. However, the impact of different types of microenvironments on the phenotypes of PLC generated by distinct oncogenes is still unclear. In addition, the cell origin of different liver cancers have not been clarified, as far as we know. Recent researches show that mature hepatocytes retain phenotypic plasticity to differentiate into cholangiocytes. More importantly, our results initially demonstrated that HCC, ICC, and cHCC-ICC could originate from mature hepatocytes rather than liver progenitor cells (LPCs), hepatic stellate cells (HSCs) and cholangiocytes in AKT-driven, AKT/NICD-driven and AKT/CAT-driven mouse PLC models respectively by using hydrodynamic transfection methodology. Therefore, liver tumors originated from mature hepatocytes embody a wide spectrum of phenotypes from HCC to CC, possibly including cHCC-ICC and HB. However, the underlying mechanism determining the cancer phenotype of liver tumors has yet to be delineated. In this review, we will provide a summary of the possible mechanisms for directing the cancer phenotype of liver tumors (i.e., ICC, HCC, and cHCC-ICC) in terms of oncogenic driver genes and tumor microenvironment. Moreover, this study initially revealed the cell origin of different types of liver cancer.

## Facts

- Liver tumor phenotype is defined by a combination of driving oncogenes but also the types of tumor microenvironments.
- Necroptosis-associated hepatic microenvironment facilitates formation of ICC, whereas apoptosis-associated hepatic microenvironment promotes formation of HCC.
- HCC, ICC, and cHCC-ICC could originate from mature hepatocytes in mouse models by using hydrodynamic transfection methodology.

## Open questions

- In addition to mature hepatocytes, it is not clear whether liver cancer could originate from hepatic progenitor cells, hepatic stellate cells and bile duct cells.
- It’s uncertain whether the formation of cHCC-ICC was jointly caused by necroptosis environment and apoptosis environment, which needs to be verified in the future.
- In the course of chemoembolization therapy for patients with HCC, a phenotypic transition from HCC to ICC was observed. The possible mechanism may lie in the necroptosis-associated hepatic microenvironment caused by chemoembolization therapy, suggesting the cell environment may directly affect the choice of treatment methods.

## Introduction

Primary liver cancer (PLC) is the fifth most prevalent cancer and the third common cause of cancer-related mortality worldwide^1^. PLC is insensitive to various treatments, which could be partly explained by its wide genetic variations, reflecting in the diverse phenotypes and histological characters. PLC may be mainly classified as the following four types: hepatocellular carcinoma (HCC), intrahepatic cholangiocarcinoma (ICC), hepatoblastoma (HB), and combined HCC and intrahepatic cholangiocarcinoma (cHCC-ICC)^2^. cHCC-ICC, as an intermediate variant of PLC, has attracted more and more attention in recent years. cHCC-ICC is described as demonstrating histologic features of both hepatocellular and biliary epithelial differentiation^3^. It is a rare primary liver malignancy, accounting for 1–14.2% of cases. However, the cell origin of PLC is still controversial.

From morphological and pathological perspectives, HCC and ICC were previously considered to originate from hepatocytes and cholangiocytes, respectively^4^. In addition, some subtypes of HCC with fetal hepatoblasts features are thought to arise from hepatic progenitor cells, which may differentiate into hepatocytes and bile duct epithelial cells under certain stimuli^5^. Generally, the cell origin of PLC may be derived from the following four types of cells: hepatocytes, cholangiocytes, hepatoblasts, and liver stem/progenitor cells. However, the cell origin of PLC and the underlying mechanism for the phenotypic determination remains unclear. Recently, some well-established lineage-tracing mouse experiments have demonstrated that HCC or ICC originates from mature hepatocytes rather than liver progenitor cells (LPCs), hepatic stellate cells (HSCs), and cholangiocytes. For example, one study showed that ICC could originate from hepatocytes in mice when the PI3K-AKT and Notch pathways were coactivated^6^. On a similar note, Mu et al. demonstrated that hepatocytes represent the cell of origin for HCC in mice. Moreover, for the subtype of HCC with a progenitor signature, it does reflect progenitor origin, but dedifferentiation of hepatocyte-derived tumor cells^7^. Therefore, liver tumors originated from mature hepatocytes consist of a wide spectrum of phenotypes from HCC to CC, possibly encompassing cHCC-ICC and HB^4^.

In this review, we will summarize the potential mechanisms for determining the cancer phenotype of hepatocyte-derived mouse liver tumors, including ICC, HCC, and cHCC-ICC, in terms of oncogenic driver genes and tumor microenvironment by combining our previous work and the latest research progress. More importantly, it may help us screen of innovative therapeutic approaches against this deadly malignancy in the future.

## Regulatory molecules and tumor microenvironment that commit ICC formation

Previous studies have shown that ICC may originate from the cells lining the bile ducts, biliary duct cells (BDCs) or liver stem/progenitor cells^8^. Nevertheless, recent studies have demonstrated that mature hepatocytes possess a capacity for cholangiocytes transdifferentiation under certain conditions^9^. For example, Nishikawa et al.^10^ found that cultured hepatocytes expressed several bile duct markers including cytokeratin (CK) 19 in a three-dimensional organoid culture system, which containing insulin and epidermal growth factor. Likewise, Michalopoulos et al.^11^ showed that hepatocytes can transdifferentiate into BDCs and help repair the damaged biliary epithelium when its proliferative capacity is being compromised. Moreover, a recent study also showed that mature hepatocytes exhibited the bile duct-like phenotype after chronic liver injury both in vivo and in vitro^9^. In addition, the notion that cell origin of ICC is mature hepatocytes was subsequently confirmed in another chemically induced ICC mouse model^12^, as well as a study by electroporating oncogenic transposon plasmids into the left liver lobe of mice^13^. Recent studies have shown a significant difference between the primary BDCs and the hepatocytes transdifferentiated BDCs. Morphologically, these hepatocytes transdifferentiated BDCs are not mature cholangiocytes with reserve for hepatocyte differentiation. Functionally, hepatocyte-derived ductules are not conducive to bile drainage. Importantly, a recent study demonstrated that these hepatocytes transdifferentiated BDCs are transcriptionally distinct from the primary BDCs as shown by RNA-sequencing analysis and ultrastructural analysis. Interestingly, these hepatocytes transdifferentiated BDCs keep their origin memory and could revert back to hepatocytes upon cessation of injury, which reflecting an adaptive injury escape mechanism^14^. Mechanistically, TGFβ signaling has been identified associated with the formation of the transdifferentiated BDCs from hepatocytes.

**Fig. 1:**
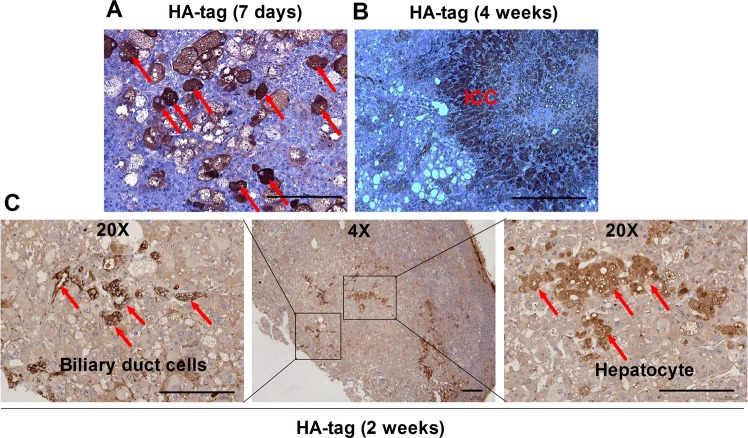
ICC could originate from hepatocytes. **a** Immunohistochemical staining (IHC) showed that some scattered HA-tag strongly positive hepatocytes were detected in AKT/NICD-injected livers after 7 days. **b** IHC results showed that HA-tag protein was also expressed in ICC tissues after 4 weeks. **c** IHC results showed that some HA-stained hepatocytes and biliary duct cells appeared after 2 weeks. ICC: intrahepatic cholangiocarcinoma (scale bars, 50 μm).

**Fig. 2:**
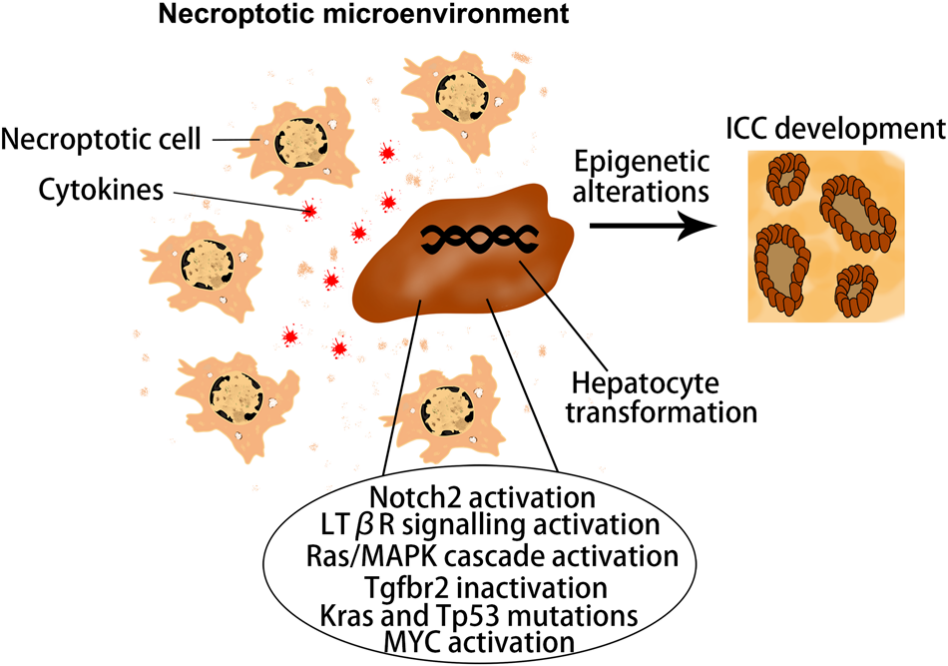
Schematic representation of regulatory molecules and tumor microenvironment that commit hepatocyte-derived ICC formation. ICC: intrahepatic cholangiocarcinoma.

In our previous research, we applied hydrodynamic tail vein injection of hemagglutinin (HA) tagged AKT and NICD plasmid (AKT/NICD), along with Sleeping Beauty (SB) plasmids into BALB/c mice (6–8 weeks) to initiate ICC development. After 7 days, some scattered HA-tag strongly positive hepatocytes were detected in AKT/NICD-injected livers (Fig. 1a). After 4 weeks, immunohistochemical results showed that HA-tag protein was also expressed in ICC, indicating that ICC could originate from these HA-tag positive hepatocytes (Fig. 1b) After 2 weeks, we found that some HA-stained hepatocytes and BDCs appeared at the same time, which further proved the hepatocytes transdifferentiation (Fig. 1c). Accordingly, cellular reprogramming- transition from hepatocyte towards a more ICC-like phenotype might be induced by genetic and cellular alterations occurring during tumorigenesis. Based on the latest research, mechanism underlying hepatocyte-derived ICC formation can be summarized as the following aspects (Fig. 2).

### Regulatory molecules that commit ICC formation

#### Notch signaling pathway

Accumulating evidence suggests that the canonical Notch cascade controls hepatocyte-derived ICC formation in mice^12,14^. Notch is a highly conservative signaling pathway that regulates cell proliferation and differentiation and plays an important role in embryonic development and in cell fate determination^15^. By using well-established lineage-tracing mouse models, Yanger et al.^16^ report that the activation of Notch is sufficient to reprogram hepatocytes into biliary epithelial cells under injury conditions that provoke a biliary response. Likewise, Fan et al.^6^ and Sekiya et al.^12^ also show that ICC can originate from fully differentiated hepatocytes by using a mouse model of hepatocyte fate tracing. However, which Notch receptor is responsible for hepatocyte-derived ICC formation appears to be more important. Most recently, a new study reveals that Notch2, rather than Notch1, controls hepatocyte-derived ICC formation in mice. In this study, Chen et al. established a murine hepatocyte-derived ICC model by co-expression of AKT and Yap plasmids in mice liver^14^. They demonstrated that deletion of Notch2 skews AKT/Yap-induced ICC pathology towards a more hepatocellular adenoma-like phenotype. However, deletion of Notch1 in tumor cells does not affect the histological type^14^. Therefore, endogenous Notch signaling is required for hepatocyte-derived ICC. This finding suggested that Notch2 could serve as a target for treatment of this deadly disease, which has great impact on clinical practice in the foreseeable future.

#### Kras and Tp53 mutations

The recent study of Hill et al.^17^ indicates Kras and Tp53 mutations facilitate formation of hepatocyte-derived ICC in the context of chronic liver injury. By using *Alb-Cre;Kras*^*LSL-G12D*^*;Tp53*^*f/f*^ transgenic mice that targeting Kras and Tp53 mutations to the mouse liver, Hill et al.^17^ demonstrated that selective induction of Kras and Tp53 mutations in mature hepatocytes in the setting of liver injury, such as DDC-induced chronic inflammation (3,5-diethoxycarbonyl-1,4-dihydrocollidine), could drive rapid progression of ICC^17^. More importantly, Tp53 has been identified as a key regulator in enabling hepatocyte-derived ICC in this context. Indeed, Tp53 has been shown to control plasticity in a number of different cellular contexts^18^ and thus Tp53 mutations may facilitate such transdifferentiation events that are implicated in hepatocyte-derived ICC pathogenesis^19^.

#### Tgfbr2

Another study reported that Tgfbr2 (TGF-β receptor II) restricts hepatocyte-derived ICC^20^. TGF-β pathway is closely related to the development of hepatic fibrosis both in mice and patients^21^. It is noteworthy that recent exon sequencing revealed a high frequency of mutations in Smad4, a key downstream mediator of TGF-β signals, in human cholangiocarcinoma^22^. Most recently, a new study reveals that hepatocyte-specific deletion of Tgfbr2 and PTEN mediated by AAV8-TBG-Cre promoted hepatocyte-derived ICC formation and reduced survival of mice^20^. Mechanistically, deletion of Tgfbr2 promotes the proliferation of cholangiocyte rather than hepatocytes, suggesting the pivotal role of epithelial Tgfbr2 in restricting cholangiocyte proliferation^20^. Although targeting TGF-β may be clinically effective for liver fibrosis, this approach may increase the risk of ICC, which needs to be paid enough attention in clinic.

#### c-Myc

The recent study of Hill et al.^23^ indicates c-Myc is required for hepatocyte-derived ICC in AKT/Fbxw7ΔF mice. The ubiquitin ligase F-box and WD repeat domain-containing 7 (FBXW7) plays an anti-cancer role in many cancers, such as HCC, colorectal cancer and gastric cancer^24^. It can lead to the degradation of several oncogenes, such as c-MYC and YAP^25^. Wang et al.^23^ generated a ICC mouse model by co-expression of Fbxw7ΔF (a dominant negative form of Fbxw7) and AKT plasmids in mice livers. Using lineage tracing technology, they confirmed that ICC lesions induced by AKT/Fbxw7ΔF derived from hepatocytes. Interestingly, selected deletion of c-Myc, as for the downstream targets of FBXW7, completely suppresses hepatocyte-derived ICC formation in AKT/Fbxw7ΔF mice^23^. Furthermore, in human ICC specimens, the expression level of Fbxw7 was negatively correlated with the transcription activity of c-myc^34^. Therefore, c-Myc could serve as a therapeutic target for ICC treatment, especially with respect to patients with low FBXW7 expression.

#### Ras/MAPK cascade

Ras/MAPK cascade may influence the formation of hepatocyte-derived ICC by promoting cell proliferation and regulating tumor microenvironment^26^. Previous studies have shown that Ras/MAPK pathway is significantly activated in human ICC^27^. In a recent study, Wang et al. generated a hepatocyte-derived ICC mouse model by hydrodynamic tail vein injection of AKT and YapS127A plasmids in mice livers^26^. They found that inhibition of Ras/MAPK cascade significantly delayed the progression of AKT/YapS127A-induced ICC. On the one hand, Ras/MAPK cascade can significantly promote the proliferation of cholangiocarcinoma cells. On the other hand, this cascade can recruit activated hepatic stellate cells (AHSC) and create hypoxic microenvironment in tumor tissues, which is key features of human ICC^28^. Because MEK is a key player in Ras/MAPK pathway, MEK inhibitors may be a therapeutic option for ICC in future clinical trials.

#### LTβR signaling

The recent study of Scarzello et al.^29^ indicates LTβR signaling accelerates formation of hepatocyte-derived ICC in AKT/β-catenin and AKT/NICD mouse models. LTβR is a member of the tumor necrosis factor (TNF) superfamily of receptors^30^ and implicated in the initiation of liver cancer^31^. AKT/CAT-induced tumors display multiple pathological features, including lipogenic hepatic foci, HB/HCC-like nodules and ICC-like lesions^32^, among which the first two types of pathological features are most common, while ICC-like lesions are relatively rare. However, when using LTβR agonists, more ICC-like tumors were observed in AKT/β-catenin mouse model, suggesting LTβR signaling skews AKT/β-catenin pathology towards a more ICC-like phenotype^29^. In addition, a role for LTβR signaling in promoting the progression of ICC was further confirmed using AKT/NICD-initiated ICC model. In preclinical and clinical research study of liver cancer, combination therapies are being widely explored and are attracting more attention increasingly. Immune agents blocking the activity of LTβR in combination with other drugs, such as Akt or β-catenin pathway inhibitor, may achieve better therapeutic effect in ICC.

### Tumor microenvironment that commits ICC formation

The so-called tumor microenvironment has been recognized as an important regulator in the initiation, development and treatment of various cancers. Recently, it has been found that necroptosis-associated hepatic cytokine microenvironment facilitates formation of ICC^33^. Tumor microenvironment is a complex environment for the survival and development of cancer cells, which mainly consists of cellular and non-cellular components^34^. Both components play a supporting role in the growth of tumors^35^. Very recently, it has been found that the microenvironment of cancer cells (especially the special form of cell death occurring in this environment) has a decisive influence on whether HCC or ICC occurs^33^. In necrotic apoptosis, a large number of cytokines are secreted from immune cells that are activated by damage-associated molecular patterns (DAMPs), which released from necroptosically dying hepatocytes^36^. While in apoptosis, vesicles are cleared by the immune system and there is no large amount of cytokine production in microenvironment^37^. Researchers found that hepatocytes with aberrantly activated oncogenes, if the cell death in their environment is caused by apoptosis, will give rise to HCC; on the other hand, if the cell death is caused by necroptosis, it will lead to ICC. These results were further validated in mouse models and human tissue samples^33^. Most importantly, the microenvironment formed by different apoptotic pathways had a great influence on two epigenetic regulators (Tbx3 and Prdm5), which are the key regulator in determining lineage commitment in liver cancer^38^. Interestingly, simultaneous Prdm5 overexpression and Tbx3 knockdown resulted in the development of ICC; however, Tbx3 overexpression combined with Prdm5 knockdown lead to the development of HCC^33^. There is evidence that inflammatory cytokines and infiltrated immune cells may play an important role in the formation of ICC because they may connect the bridge between the oncogenic driver genes and hepatic death. Seehawer et al.^33^found that some specific cytokines (e.g., Ccl4, Ccl8, Osm, Ccl6, Cxcl13, Pf4, and Aimp1) are secreted by immune cells, which are activated by DAMPs released from necroptosically dying hepatocytes^33^. These specific cytokines may act on hepatocytes together with aberrantly activated oncogenes and further led to ICC. However, they also found that the infiltrated immune cells (e.g., T cells, monocytes and (neutrophilic) granulocytes as well as B cells and antigen-presenting cells) were not obvious in the necroptosic microenvironment, suggesting the limited role of infiltrated immune cells in ICC formation^33^. In the future, in addition to the types of cancer, we should also study whether the tumor microenvironment directly affects the choice of treatments. In the course of chemoembolization therapy for patients with HCC, we found that some primary HCC could transform into ICC. The possible mechanism may lie in the necroptosis-associated hepatic cytokine microenvironment caused by chemoembolization therapy, which may promote a phenotypic transition from HCC to ICC. This may be one of the important reasons for drug resistance in patients with liver cancer.

## Regulatory molecules and tumor microenvironment that may commit cHCC-ICC formation

cHCC-ICC is described as demonstrating histologic features of both hepatocellular and biliary epithelial differentiation. It is a rare primary liver malignancy, accounting for 1% to 14.2% of cases^39^. The diagnosis of cHCC-ICC is usually made at pathologic evaluation after either resection or transplantation and it is practically impossible to achieve an accurate, pre-operative diagnosis of cHCC-ICC with tumor markers or abdominal imaging^40^. Accordingly, the actual incidence may be higher due to frequent difficulty in accurate pathological assessment. Although little is known clinically about this type of malignancy, the data available indicate that it is aggressive and likely signify a unique subset of PLCs, which merits clinical distinction^41^. Recent lineage tracing experiments in mice have demonstrated that some subtypes of liver cancer, such as HCC and ICC, are derived from mature hepatocytes rather than from liver stem/progenitor cells^7^. Moreover, by analyzing systematic mutations, somatic copy number variations, and clonal analyses of human cHCC-ICC tissues, Moeini et al.^42^ and Wang et al.^43^ demonstrated that HCC and ICC components share a common cell of origin.

**Fig. 3:**
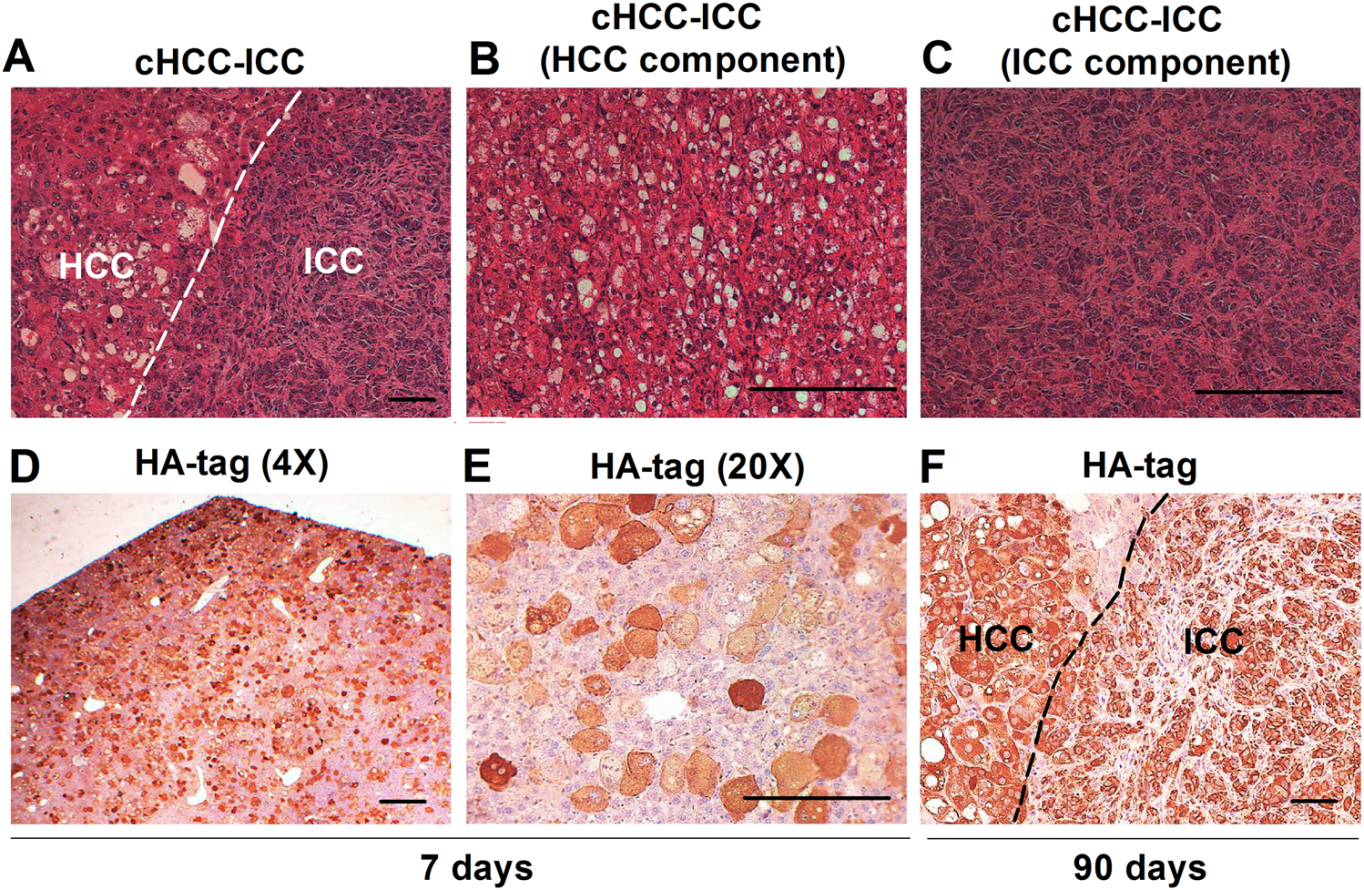
HCC and ICC components of cHCC-ICC could both originate from hepatocytes. **a**–**c** The moribund livers (5%) following 2 months of AKT and CAT injection displayed a pathological characteristics of cHCC-ICC containing both HCC and ICC two components. **d**, **e** IHC showed that some scattered HA-tag strongly positive hepatocytes were detected in AKT/CAT -injected livers after 7 days. **f** IHC showed that HA-tag protein was also expressed in both HCC and ICC two components of cHCC-ICC tumor tissues after 90 days. HCC: hepatocellular carcinoma; ICC: intrahepatic cholangiocarcinoma; cHCC-ICC: combined hepatocellular carcinoma and intrahepatic cholangiocarcinoma; IHC: immunohistochemical staining (scale bars, 50 μm).

Consistently, our previous studies have shown that cHCC-ICC may originate from hepatocytes in AKT/CAT model. AKT/CAT-initiated tumors display multiple pathological characteristics, including lipogenic hepatic foci, HCC, ICC and cHCC-ICC. We found the moribund livers (5%) following 2 months of AKT and CAT injection displayed a pathological characteristics of cHCC-ICC containing both HCC and ICC two components (Fig. 3a–c). Some scattered HA-tag strongly positive hepatocytes were detected in AKT/CAT -injected livers after 7 days (Fig. 3d, e). Interestingly enough, after 90 days, IHC results showed that HA-tag protein was also expressed in both HCC and ICC two components of cHCC-ICC tumor tissues (Fig. 3f), indicating that HCC and ICC components might originate from these HA-tag positive hepatocytes.

**Fig. 4:**
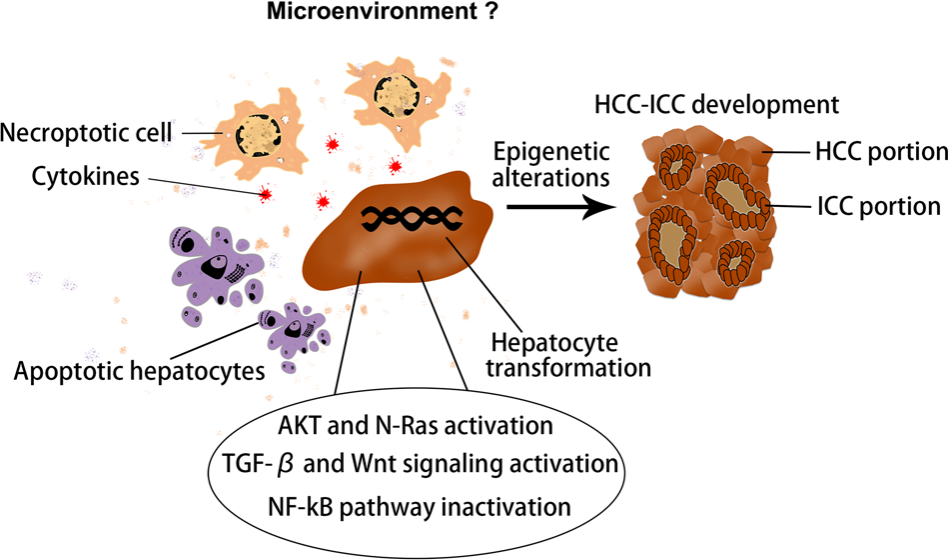
Schematic representation of regulatory molecules and tumor microenvironment that may commit hepatocyte-derived cHCC-ICC formation. cHCC-ICC: combined hepatocellular carcinoma and intrahepatic cholangiocarcinoma.

Therefore, liver tumors originated from mature hepatocytes consist of a wide spectrum of phenotypes from HCC to CC, possibly encompassing cHCC-ICC^4^. Based on the latest research, mechanism underlying hepatocyte-derived cHCC-ICC formation can be summarized as the following aspects (Fig. 4).

### TGF-β, Wnt, and Notch signaling

**Fig. 5:**
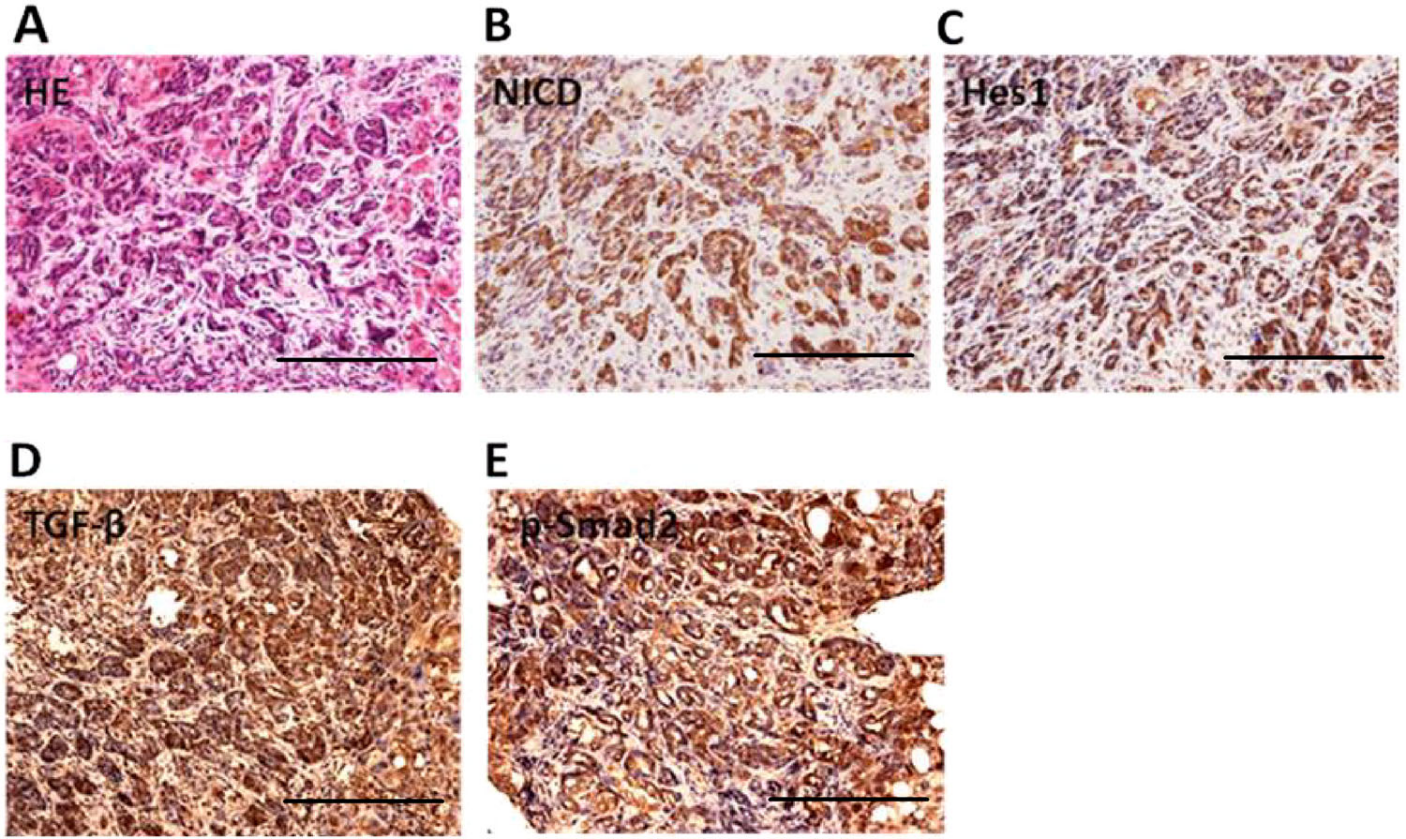
IHC showed that TGF-β and Notch signalings were activated in the ICC components of human cHCC-ICC. ICC: intrahepatic cholangiocarcinoma; cHCC-ICC: combined hepatocellular carcinoma and intrahepatic cholangiocarcinoma; IHC: immunohistochemical staining (scale bars, 50 μm).

TGF-β, Wnt/β-catenin and Notch signalings were identified as the major signaling activated in human cHCC-ICC specimens^41^. Indeed, by using a genome-wide transcriptional analysis, Coulouarn et al.’s^44^ study showed that cHCC-ICC exhibited a gene signature characteristic of the activation of the Wnt/β-catenin pathway, which is closely related to the development of bile duct morphology. Interestingly, TGF-β signaling pathway has been reported to be activated in cHCC-ICC and could be attributed to the presence of the tumoral fibrous stroma with a cholangiocarcinoma-like gene expression trait^45^. Such results are in accord with a previous study published in Nature, suggesting that TGF-β signaling enhances the formation of the biliary system from hepatocytes through a transdifferentiation mechanism^46^. Therefore, TGF-β and Wnt/β-catenin pathway may be involved in the formation of ICC components in cHCC-ICC. In addition, mutations of TERT promoter and TP53, as well as substantial intratumoral heterogeneity, often appear in cHCC-ICC^41^. Consistently, our previous studies have shown that TGF-β and Notch signalings were activated in human cHCC-ICC, especially in the ICC components (Fig. 5).

### NF-kB pathway

The function of NF-κB in liver cancer is still contradictory. Some studies have shown that the NF-κB pathway promotes inflammation-related cancer^47^, whereas inhibition of NF-κB activity in hepatocytes promotes the spontaneous formation of HCC, indicating that the NF-κB pathway function as tumor suppressor in hepatocytes^48^. A recent study, the first to analyze the role of NF-kB pathway in the progression of cHCC-ICC, indicates that block of NF-kB signaling skews c-Myc-driven HCC pathology towards a more cHCC-ICC-like phenotype^49^. It was well known that the tumor phenotype induced by c-Myc often manifests as HB^50^, however, ICC has not been reported previously. Importantly, after inhibition of NF-kB pathway, an additional tumor component resembling ICC was observed in this model, which was accompanied by MAPK activation, reflecting previous reports on the critical role of NF-kB pathway in cholangiocarcinoma^51^. Accumulating evidence suggests that liver cancer phenotype can be influenced by sequential oncogenic dysregulation and the inflammatory milieu^29^. Given NF-κB deletion led to an increase in infiltrating inflammatory cells^52^, it is reasonable that the chronic inflammatory environment caused by NF-κB ablation may modulate the phenotypic transition in this model.

### AKT and N-Ras (N-Ras-V12, a persistently active form of N-Ras)

Activation of AKT and Ras pathways is often implicated in hepatocarcinogenesis. A previous study showed that overexpression of AKT and N-Ras in the mouse liver (AKT/Ras) by way of hydrodynamic gene transfer can accelerate both HCC and ICC development (i.e., cHCC-ICC), with ICC lesions accounting for about 10% of the total lesion area^53^. Mechanistically, mTORC1, FOXM1/SKP2, and c-Myc signaling cascades were found to be involved in the mediating AKT/N-Ras-induced hepatocarcinogenesis^54^. In addition, N-Ras-V12 oncogene was delivered to the livers of p19Arf-null or heterozygous mice to elicit tumor formation. The results showed that the tumor pathological type of this model was cHCC-ICC, further suggesting a key role of N-Ras-V12 in the development of cHCC-ICC^55^.

### Tumor microenvironment that may commit cHCC-ICC formation

According to our knowledge, there is no report on the tumor microenvironment of cHCC-ICC so far. As mentioned earlier, necroptosis-associated hepatic cytokine microenvironment facilitates formation of hepatocyte-derived ICC, whereas apoptosis-associated hepatic cytokine microenvironment promotes formation of hepatocyte-HCC. Based on this, we speculate that hepatocytes with aberrantly activated oncogenes, if the cell death in their environment is jointly caused by necroptosis and apoptosis, will give rise to cHCC-ICC (Fig. 4). However, this viewpoint needs to be verified by experiments in the future.

## Regulatory molecules and tumor microenvironment that commit HCC formation

Until recently, some well-established lineage-tracing mouse experiments have further demonstrated that HCC originates from mature hepatocytes rather than LPCs, hepatic stellate cells (HSCs) and biliary compartment both in genotoxic and genetic models^7^. In order to study the molecular mechanism of hepatocyte-derived HCC formation, various primary HCC mouse models were established (Table 1). For instance, mouse HCC induced by CCl4, diethylnitrosamine (DEN), or aristolochic acid was often accompanied with reactivation of a variety of fetal liver genes, such as Gpc3, Afp, Slpi, Spink3, and Abcd2^56–58^. Moreover, various transgenic mouse models of HCC have been successfully generated by overexpression of oncogenes such as AKT, Myc, Bmi1, c-Met, Tgfa, E2F1, Ccnd1, Spry2Y55F, and HRASG12V, or genes that encode viral proteins, such as HbsAg, HBX, and SV40 T-Ag (Table 1)^59–77^. However, these transgenic mouse models have several limitations, such as high costs, time consuming and requiring high professional knowledge and skills. Hydrodynamic gene delivery is a new method that combines with the SB mediated somatic integration for long-term gene expression in mouse hepatocytes, which has been used in developing novel murine models for HCC (Table 1)^4,6,23,32,33,49,54,55,78–87^. Through this technique, Che et al.^88^ reveals a novel crosstalk between aberrant lipogenesis and cholesterol biosynthesis pathways in the progression of HCC. Shang et al.^89^ demonstrated that co-overexpression of focal adhesion kinase (FAK) and β-Catenin leads to HCC formation. Therefore, hydrodynamic transfection is a reliable method to induce liver tumor and can be used to study the role of genes with unknown functions in hepatocarcinogenesis.

**Table 1:** The various mouse models of liver cancer.

Genes	Tumor type	Mouse strains	Latency	Reference
*Genetically engineered mouse models for liver cancer*				
AAT	HCC	Transgenic mice using human alpha 1-antitrypsin M and Z genomic clones	52–90 weeks	Geller et al.^60^
NEMO^−/−^	HCC	NEMO^Δhepa^ mice	52 weeks	Beraza et al.^61^
PTEN^−/−^	HCC	PTEN-deficient mice	42–44 weeks	Watanabe et al.^62^
PTEN^−/−^ + GRP94^−/−^	cHCC-ICC	PTEN and GRP94 two liver-specific knockout mouse	25 weeks	Chen et al.^63^
HCV core	HCC	Transgenic for the HCV core gene	80–105 weeks	Moriya et al.^64^
TAK1^−/−^	HCC	Tak1^Δhepa^ mice	39 weeks	Inokuchi et al.^65^
HBx	HCC	Transgenic mice expressed HBV-encoded gene products	52–104 weeks	Chisari et al.^66^
KRAS^G12D^ + HBx	HCC	Kras(G12D) and HBx double transgenic mice	34 weeks	Ye et al.^67^
c-myc	HB	c-myc single transgenic mice	65–90 weeks	Thorgeirsson et al.^68^
c-myc + EGF	HCC	Autocrine growth factor IgEGF and c-myc single transgenic mice	12–18 weeks	Tönjes et al.^69^
c-myc + E2F1	HCC	c-Myc/E2F1 transgenic mouse	26–39 weeks	Calvisi et al.^70^
P53^−/−^ + c-myc	HCC	c-Myc/p53KO mice	21 weeks	Klocke et al.^71^
P53^−/−^	HCC	P53^Δhepa^ mice	60 weeks	Katz et al.^72^
EGF	HCC	Autocrine growth factor IgEGF transgenic mice	24–36 weeks	Tönjes et al.^69^
SV40 T-antigen	HCC	Mice expressing SV 40 early sequences	20 weeks	Lou et al.^73^
E2F-1	HCC	E2f1 transgenic mice	52 weeks	Lee et al.^74^
APC^−/−^	HCC	APC^Δhepa^ mice	38 weeks	Colnot et al.^75^
TGF- a	HCC	TGF-alpha transgenic mice	>52 weeks	Lee et al.^76^
β-catenin(Dex3) + HRASG12V	HCC	Mouse strain containing a mutant beta-catenin allele of which exon 3 was sandwiched by loxP sequences [Catnb(lox(ex3))]	8 weeks	Harada et al.^77^
*Application of the hydrodynamic transfection methodology to induce liver cancer*				
myr-AKT	HCC	C57BL/6J, FVB/N	6 months	Calvisi et al.^78^
myr-AKT and DN90-b-catenin	HCC	C57BL/6J, FVB/N	1 months	Calvisi DF et al.^79^
myr-AKT and NRasV12	cHCC-ICC	C57BL/6J, FVB/N	1 months	Ho et al.^54^
myr-AKT and NICD	ICC	C57BL/6J, FVB/N	3 weeks	Fan et al.^6^
c-Met and DN90-b-catenin	HCC	C57BL/6J, FVB/N	3 months	Tward et al.^80^
NRasV12 and DN90-b-catenin	HCC	C57BL/6J, FVB/N	3 months	Lee et al.^81^
NEMO (IKKγ) KO + c-Myc	cHCC-ICC	C57BL/6J, FVB/N	45 days	He et al.^49^
Myc and human NRASG12V	HCC	p19Arf^−/−^	4 weeks	Seehawer et al.^33^
mouse Myc and Akt1	HCC	p19Arf^−/−^	4 weeks	Seehawer et al.^33^
FAK and DN90-b-catenin	HCC	57BL/6J, FVB/N	24 weeks	Shang et al.^82^
myr-AKT and c-Myc	HCC	57BL/6J, FVB/N	8 weeks	Yamamoto et al.^4^
myr-AKT/c-Myc/YAP	HCC	57BL/6J, FVB/N	3 weeks	Yamamoto et al.^4^
myr-AKT and YAP	ICC	57BL/6J, FVB/N	6 weeks	Yamamoto et al.^4^
c-Myc and YAP	HB	57BL/6J, FVB/N	16 weeks	Yamamoto et al.^4^
NICD1	ICC	57BL/6J, FVB/N	5 months	Fan et al.^6^
HRasV12 and shP53	Undifferentiated liver tumors	57BL/6J, FVB/N	1 week	Ju et al.^83^
NRasV12	cHCC-ICC	Ink4A/Arf^−/−^	7 weeks	Carlson et al.^55^
myr-AKT and Spry2Y55F	HCC	57BL/6J, FVB/N	4 months	Wang et al.^84^
c-Myc and shp53	HCC	57BL/6J, FVB/N	7 months	Ju et al.^85^
AKT/Fbxw7ΔF	ICC	57BL/6J, FVB/N	10 weeks	Wang et al.^23^
Nras-FAH and shP53	HCC	Fah^−/−^	10 weeks	Wangensteen et al.^86^
Bmi1 and NRasV12	HCC	57BL/6J, FVB/N	6 months	Xu et al.^87^
*Application of the chemical carcinogens to induce liver cancer*				
Diethylnitrosamine (DEN)	HCC	57BL/6J, FVB/N	14 months	Ngo et al.^57^
Aristolochic acid	HCC, cHCC-ICC	57BL/6J, FVB/N	In a dose-dependent manner	Lu et al.^58^

**Fig. 6:**
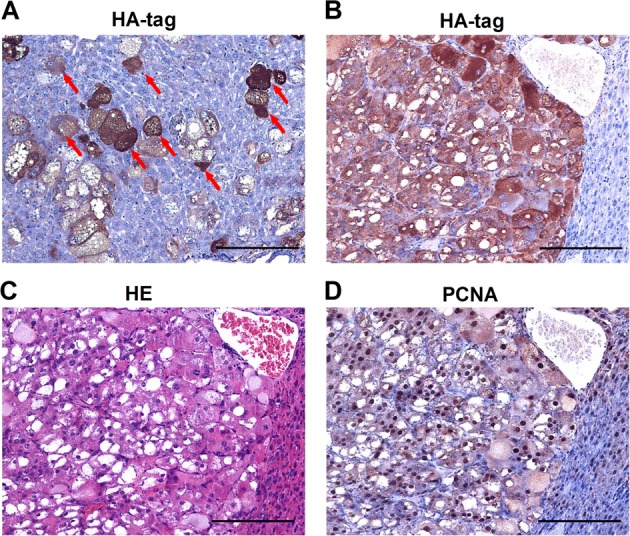
HCC could originate from hepatocytes. **a** IHC showed that some scattered HA-tag strongly positive hepatocytes were detected in AKT-injected livers after 7 days. **b** IHC results showed that HA-tag protein was also expressed in HCC tumor tissues after 6 months. **c** HE staining showed that AKT-initiated tumors were characterized by lipid rich droplets. **d** IHC results showed that PCNA protein was highly expressed in HCC tumor tissues after 6 months. HCC: hepatocellular carcinoma; IHC: immunohistochemical staining; HE: hematoxylin-eosin staining (scale bars, 50 μm).

**Fig. 7:**
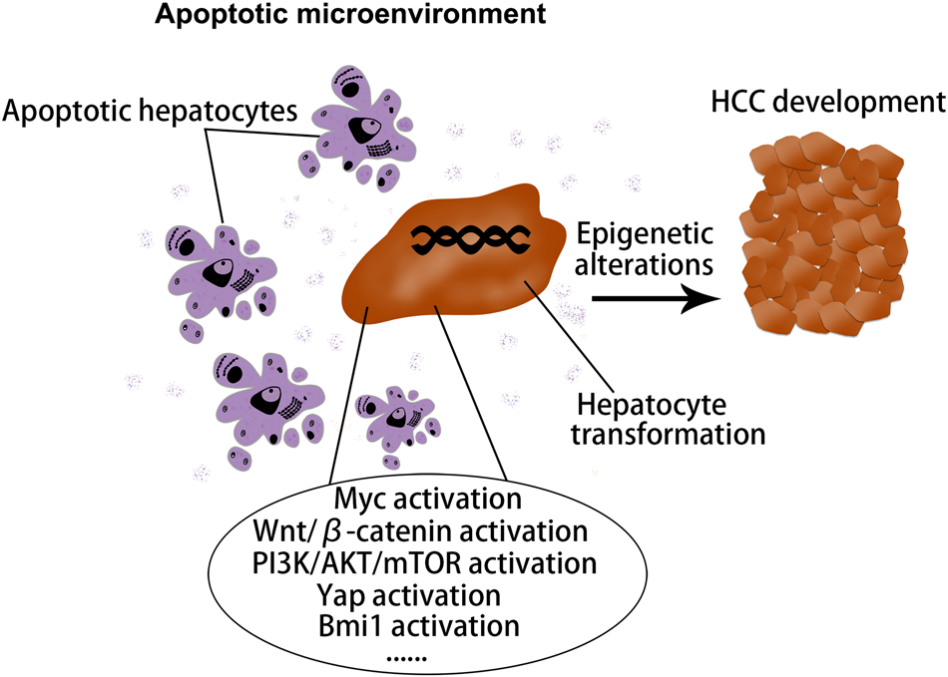
Schematic representation of regulatory molecules and tumor microenvironment that may commit hepatocyte-derived HCC formation. HCC: hepatocellular carcinoma.

Consistently, our previous studies have shown that HCC may originate from hepatocytes in AKT mouse model. AKT-initiated tumors were characterized by lipid rich droplets and high proliferation (Fig. 6c, d). Some scattered hepatocytes with strongly positive HA-tag were detected in AKT-injected livers after 7 days (Fig. 6a). IHC results showed that HA-tag protein was also expressed in HCC tumor tissues after 6 months (Fig. 6b), indicating that HCC might originate from these HA-tag positive hepatocytes. Based on the latest research, mechanism underlying hepatocyte-derived HCC formation can be summarized as the following aspects (Fig. 7).

### Hepatocarcinogenesis due to the interaction of multiple genes

The occurrence of HCC is a complex process accompanied by the activation of multiple signaling pathways, which plays a synergistic role in the process of tumorigenesis^90^. Numerous studies have confirmed that PI3K/AKT/mTOR pathway and Wnt/β-catenin pathways play an important role in the development of HCC^91^. For instance, hydrodynamical codelivery of activated forms of AKT (pT3-EF1α-HA-myr-AKT) and β-catenin (pT3-EF1α-Δ90β-catenin, CAT) oncogenes into mouse livers using the SB transposon system efficiently and rapidly induces primary hepatic tumors. AKT/CAT-initiated tumors display multiple pathological characteristics, including early lipogenic hepatic foci and subsequent HB/HCC-like nodules, which is rich in lipids^29^. Importantly, this provides a good animal model for the study of steatosis-related liver cancer. In addition, the activated form of AKT was found to cooperate with activated Myc, Yap, NRasV12 or Spry2Y55F pathways to induce HCC formation in the mouse^84^. Hydrodynamical codelivery of the activated mutant of β-catenin and c-MET^1^ or NRasV12^81^ into mouse livers can also efficiently induce HCC over a short latency. Using the same method, Li et al.^92^ reported that the introduction of YAPS^127A^ and PIK3CA^H1047R^ (a constitutively active mutant of PI3K) induces liver cancers with many pathological features. Fan et al.^93^ found that Bmi1 is required for AKT/Ras -induced HCC development. Altogether, these results reflect the complex interaction of different oncogenes in hepatocyte-derived HCC formation.

### A single gene sufficient for hepatocarcinogenesis

Activated PI3K/Akt/mTOR pathway is closely related to poor differentiation, early recurrence and poor prognosis of HCC^94^. Four weeks after hydrodynamical delivery of AKT plasmids, the livers are pale and greasy. Microscopically, hepatocytes were abundant with cytoplasmic lipid and characterized by the intermingled small ductular structures^95^. After 22-32 weeks of transfection, all AKT mice developed lethal liver cancer. In general, the livers of AKT mice were pale and enlarged. There were many tumor nodules on the surface. Microscopically, these tumor cells were characterized by increased cell volume and transparent cytoplasm due to fat accumulation^95^. This suggests that overexpression of AKT alone is sufficient to form liver cancer. For another example, MYC oncogene has been implicated in human liver cancer^96^. It was reported that MYC was over expressed in over 70% of viral or alcohol-related human HCC^96^. Hydrodynamic transfection of MYC caused lethal burden of liver cancer by 6–8 weeks post injection. Pathologically, MYC tumors are poorly differentiated and resemble human HBs with cancer stem cells-like properties^97^. All these studies demonstrate that a single gene, such as MYC or AKT, is sufficient for hepatocarcinogenesis, even if not combined with other oncogenes.

### Tumor microenvironment that commits HCC formation

Chronic liver inflammation has been implicated in tumorigenesis. Actually, most HCC develops in an inflammatory environment caused by viral hepatitis and alcoholic or nonalcoholic steatohepatitis^98^. Recent studies have shown that inflammation microenvironment can induce transformation of tumor types. For example, Matter et al.^99^ demonstrated that chronic liver inflammation caused by DDC (3,5-diethoxycarbonyl-1,4-dihydrocollidine) changed AKT/CAT-induced tumors pathology. AKT/CAT-induced tumors were steatotic and contained lipid droplets, whereas lipid content in tumors of AKT/CAT with DDC group was decreased significantly. Pathological types of AKT/CAT-induced liver cancer can be classified into three types: hepatocellular adenoma (HCA), HCC, and HB. In AKT/CAT group, the proportion of HCC was 5–25%, while in AKT/CAT with DDC group, the proportion of HCC was 5–50%, suggesting that chronic inflammation promotes the phenotypic transition from HCA to HCC. Likewise, chronic inflammation microenvironment induced by DDC can also reduce lipid droplets in AKT-NRAS^G12V^ tumors^99^. Altogether, this illustrates that driving oncogenes and tumor microenvironment jointly determined the hepatocyte-derived HCC formation.

**Fig. 8:**
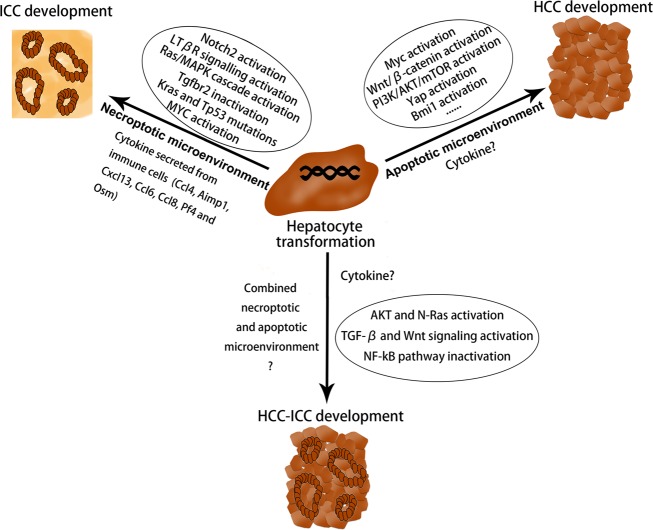
Oncogenic driver genes and tumor microenvironment determine the type of liver cancer. HCC: hepatocellular carcinoma; ICC: intrahepatic cholangiocarcinoma; cHCC-ICC: combined hepatocellular carcinoma and intrahepatic cholangiocarcinoma.

In summary, this review summarizes the possible mechanism of lineage determination in the development of PLC, including ICC, HCC, and cHCC-ICC (Fig. 8). We put forward the notion that the combined effects of oncogenic driver genes and tumor microenvironment decides the cancer phenotype of hepatocyte-derived mouse liver tumors. PLC always occurs inevitably in a variety of tumor microenvironments, in which different types of cell death such as necrosis, apoptosis or necroptosis occur. It is noteworthy that hepatocytes with aberrantly activated oncogenes will lead to ICC when cell death in their environment is caused by necroptosis with lots of cytokines production. In addition, various intracellular signaling cascades such as Notch2, MYC, Tgfbr2, and Ras/MAPK pathway in hepatocytes mediate the hepatocyte-derived ICC formation (Fig. 2). On the other hand, if the cell death in their environment is caused by apoptosis, hepatocytes with aberrantly activated oncogenes will give rise to HCC. It is well known that some classical cancer-related signalings such as MYC, Yap, Bmi1, Wnt/β-catenin, and PI3K/AKT/mTOR pathways were implicated in the hepatocyte-derived HCC formation (Fig. 7). cHCC-ICC is a rare primary liver malignancy and the incidence is increasing in the last twenty years, however, its pathogenesis is still poorly understood. Future work is needed to determine whether necroptosis, apoptosis or both occur in the tumor microenvironment that mediate the hepatocyte-derived cHCC-ICC formation (Fig. 4). In conclusion, the possible mechanism of lineage determination in the development of PLC has yet to be delineated. Deciphering the detailed roles of oncogenic driver genes and tumor microenvironment in PLC would certainly pave the way for the development of novel therapies.
